# Development of an immune-related gene pairs signature for predicting clinical outcome in lung adenocarcinoma

**DOI:** 10.1038/s41598-021-83120-4

**Published:** 2021-02-11

**Authors:** Chunlei Wu, Quanteng Hu, Dehua Ma

**Affiliations:** Department of Thoracic Surgery, Taizhou Hospital, No. 150 Ximen Street, Linhai, Taizhou, Zhejiang China

**Keywords:** Lung cancer, Tumour immunology, Computational models

## Abstract

Lung adenocarcinoma (LUAD) is the main pathological subtype of Non-small cell lung cancer. We downloaded the gene expression profile and immune-related gene set from the TCGA and ImmPort database, respectively, to establish immune-related gene pairs (IRGPs). Then, IRGPs were subjected to univariate Cox regression analysis, LASSO regression analysis, and multivariable Cox regression analysis to screen and develop an IRGPs signature. The receiver operating characteristic curve (ROC) was applied for evaluating the predicting accuracy of this signature by calculating the area under ROC (AUC) and data from the GEO set was used to validate this signature. The relationship of 22 tumor-infiltrating immune cells (TIICs) to the immune risk score was also investigated. An IRGPs signature with 8 IRGPs was constructed. The AUC for 1- and 3-year overall survival in the TCGA set was 0.867 and 0.870, respectively. Similar results were observed in the AUCs of GEO set 1, 2 and 3 (GEO set 1 [1-year: 0.819; 3-year: 0.803]; GEO set 2 [1-year: 0.834; 3-year: 0.870]; GEO set 3 [1-year: 0.955; 3-year: 0.827]). Survival analysis demonstrated high-risk LUAD patients exhibited poorer prognosis. The multivariable Cox regression indicated that the risk score was an independent prognostic factor. The immune risk score was highly associated with several TIICs (Plasma cells, memory B cells, resting memory CD4 T cells, and activated NK cells). We developed a novel IRGPs signature for predicting 1- and 3- year overall survival in LUAD, which would be helpful for prognosis assessment of LUAD.

## Introduction

Lung cancer (LC) is the most common cancer globally.^1^ There was estimated that approximately 234,000 new cases were diagnosed as LC per year, which accounts for 14% and 13% new malignant tumor cases in men and women, respectively^1,2^. Additionally, LC is the main cause of cancer-related deaths and result in over 170,000 deaths annually. Non-small cell lung cancer (NSCLC) is the most common LC (85%) and lung adenocarcinoma (LUAD) is the main pathological subtype of NSCLC (50%)^2,3^. TNM staging (AJCC) is the most commonly used parameter for clinical decision and assessment of the clinical outcome in LUAD^4,5^. However, emerging studies have shown that although patients with the same TNM stage and treatment strategy, the prognosis regimen different, indicating that TNM staging alone may not provide adequate information for prognosis assessment in LC.

Recently, researchers have come to realize that the immune system plays a vital role in the development and progression of malignant tumors^6,7^. Immune cells recognize malignant cells and eradicate them through immune surveillance^8^. However, tumors could manipulate the immune system to avoid recognition of tumor-associated antigens and to facilitate their own development^9^. Based on this theory, immunotherapy which acts via harnessing the immune system against tumors has been approved for the treatment of numerous tumors and revolutionized cancer treatment.

Aberrations of gene expression are universal events in malignancies and could facilitate tumor progression^10^. Omics technology provides a novel opportunity to understand gene changes and potential mechanisms in cancers. In addition, bioinformatics analysis could secondary analyze the result of high throughput sequencing to identify new tumor biomarkers and provide more accurate prognosis prediction and clinical decision.

Immune-related gene pairs (IRGPs) signature has been established in several cancers including colorectal cancer^11^, liver cancer^12^, and ovarian cancer^13^, and shown well accurate prognosis prediction. Gene pair refers to the random pairing of one gene with other genes. Two paired genes make up a gene pair. The expression levels of two genes in a specific sample were compared in pairs. The method for gene pair was based on a relative ranking of gene expression level, which could reduce the shortcomings of gene expression data processing, such as batch effects^13,14^. In this study, we downloaded gene expression profiles from The Cancer Genome Altas (TCGA, https://cancergenome.nih.gov) and immune-related gene set from ImmPort (https://www.immport.org/home), respectively, to perform systematic and comprehensive analysis on the characteristics of IRGPs and develop an IRGPs signature in LUAD. Then, we validated the IRGPs signature with data from Gene Expression Omnibus (GEO, https://www.ncbi.nlm.nih.gov) and evaluated the predictive accuracy of the IRGPs signature by calculating the area under curve (AUC) of receiver operating characteristic curve (ROC) and c-index. Then, we compared this signature with clinical characteristics to prove the predictive accuracy and effectiveness of the IRGPs signature. Moreover, we applied the CIBERSORT algorithm to determine 22 tumor-infiltrating immune cells (TIICs) and the ESTIMATE (Estimation of Stromal and Immune cells in Malignant Tumour tissues using Expression data) algorithm to calculate immune and stromal scores, and investigated the relationship of them with IRGPs signature.

## Results

### Patient data sets

**Figure 1:**
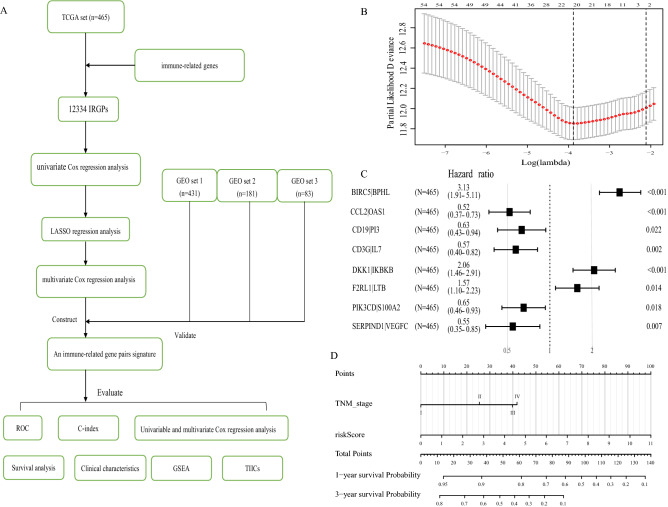
Construction of a IRGPs signature in the TCGA set. (**A**) The flow diagram of this study. (**B**) “Leave- one-out-cross-validation” for parameter selection in LASSO regression to filter out 21 IRGPs. (**C**) The forest map of multivariate Cox regression analysis to establish a IRGPs signature with 8 IRGPs. (**D**) A nomogram with the IRGPs signature and TNM stage for the prediction of 1- and 3- year overall survival. TCGA: The Cancer Genome Altas; IRGPs: Immune-related gene pairs.

A total of 1160 LUAD patients were collected, including TCGA set from TCGA database: 465 cases; GEO set 1 from GSE68465: 431 cases; GEO set 2 from GSE41271: 181 cases; GEO set 3 from GSE30219: 83 cases. All clinical information ( age, gender, smoking, histologic grade, TNM grade, tumor size, lymph node metastasis, and distance metastasis) were present as number (No.) and percentage (%) in Table 1. The flow diagram of this study was shown in Fig. 1A.

**Table 1:** The baseline characteristics of lung adenocarcinoma patients in this study.

Parameter	TCGA set	GEO set 1	GEO set 2	GEO set 3
Database	TCGA-LUAD	GSE68465	GSE41271	GSE30219
**Gender**				
Female	254 (54.62%)	216 (50.12%)	90 (49.72%)	18 (21.69%)
Male	211 (45.38%)	215 (49.88%)	91 (50.28%%)	65 (78.31%)
**Age**				
≤ 65	232 (49.89%)	226 (52.44%)	102 (56.35%)	60 (72.29%)
> 65	233 (50.11%)	205 (47.56%)	79 (43.65%%)	23 (27.71%)
**EGFR mutation**				
No	174 (37.42%)	NA	NA	NA
Yes	69 (15.05%)	NA	NA	NA
NA	221 (47.53%)	431 (100%)	181 (100%)	83 (100%)
**KRAS mutation**				
No	34 (7.32%)	NA	NA	NA
Yes	17 (3.66%)	NA	NA	NA
NA	414 (89.02%)	431 (100%)	181 (100%)	83 (100%)
**Smoking**				
Never	62 (13.33%)	48 (11.14%)	26 (14.36%)	NA
Ever	391 (84.09%)	295 (68.45%)	155 (85.64%%)	NA
NA	12 (2.58%)	88 (20.41%)	0	83 (100%)
**Radiotherapy**				
No	336 (9.38%)	353 (81.90%)	NA	NA
Yes	53 (85.62%)	64 (14.85%)	NA	NA
NA	76 (5.00%)	14 (3.25%)	181 (100%)	83 (100%)
**Chemotherapy**				
No	461 (99.14%)	329 (76.33%)	NA	NA
Yes	3 (0.64%)	89 (20.65%)	NA	NA
NA	1 (0.22%)	13 (3.02%)	181 (100%)	83 (100%)
**Histologic grade**				
Poor	NA	161 (37.35%)	NA	NA
Moderate	NA	203 (47.10%)	NA	NA
Well	NA	60 (13.92%)	NA	NA
NA	465 (100%)	7 (1.63%)	181 (100%)	83 (100%)
**TNM stage**				
I	261 (56.12%)	270 (62.65%)	100 (55.25%)	69 (83.13%)
II	106 (22.80%)	99 (22.97%)	28 (15.47%)	12 (14.46%)
III	73 (5.70%)	60 (13.92%)	49 (27.07%)	2 (2.41%)
IV	84 (18.06%)	0	4 (2.21%)	0
NA	1 (0.22%)	2 (0.46%)	0	0
**Tumor size**				
T1	159 (34.19%)	145 (33.64%)	NA	NA
T2	248 (53.33%)	244 (56.61%)	NA	NA
T3	40 (8.60%%)	27 (6.26%)	NA	NA
T4	18 (3.87%)	11 (2.55%)	NA	NA
NA	0	4 (0.94%)	181 (100%)	83 (100%)
**Lymph node**				
N0	309 (66.45%)	292 (67.75%)	NA	NA
N1–3	151 (32.47%)	137 (31.79%)	NA	NA
NA	5 (1.08%)	2 (0.46%)	181 (100%)	83 (100%)
**Metastasis**				
M0	441 (94.84%)	429 (99.54%)	NA	NA
M1	24 (5.16%)	0	NA	NA
NA	0	2 (0.46%)	181 (100%)	83 (100%)
**Survival status**				
Alive	310 (66.67%)	202 (46.87%)	112 (61.88%%)	40 (48.19%)
Dead	155 (33.33%)	229 (53.13%%)	69 (38.12%)	43 (51.81%)
**Risk score**				
Low	313 (50.11%)	334 (57.54%)	117 (64.64%)	22 (26.51%)
High	152 (49.89%)	97 (42.26%)	64 (35.36%)	61 (73.49%)
Total	465 (100%)	431 (100%)	181 (100%)	83 (100%)

*TCGA* The Cancer Genome Altas, *GEO* Gene Expression Omnibus, *NA* represents information not available.

### Construction of a prognostic IRGPs signature

A total of 12,334 IRGPs were paired. With *P* < 0.001 as the cut-off criterion, univariate Cox regression analysis identified 54 IRGPs that were highly related to the overall survival (OS) of LUAD patients. Then, 54 IRGPs were subjected to the Least Absolute Shrinkage and Selection Operator (LASSO) regression analysis with iteration = 1000, and 21 IRGPs were filtered out (Fig. 1B). Finally, with multivariate Cox regression analysis, 8 top OS-related IRGPs were identified and used to construct a prognostic IRGPs signature and develop a risk score formula (Fig. 1C; Table 2). The risk score formula was presented as follows.$$\begin{aligned} {\text{Risk}}\,{\text{score}} & = (1.139*{\text{Score}}\,{\text{BIRC5}}|{\text{BPHL}}) + ( - 0.658*{\text{Score}}\,{\text{CCL2}}|{\text{OAS1}}) \\ & \quad + ( - 0.461*{\text{Score}}\,{\text{CD19}}|{\text{PI3}}) + ( - 0.557*{\text{Score}}\,{\text{CD3G}}|{\text{IL7}}) \\ & \quad + (0.723*{\text{Score}}\,{\text{DKK1}}|IKBKB) + (0.448* {\text{Score}}\,{\text{F2RL1}}|{\text{LTB}}) \\ & \quad + ( - 0.428*{\text{Score}}\,{\text{PIK3CD}}|{\text{S100A2}}) + ( - 0.606*{\text{Score}}\,{\text{SERPIND1|VEGFC}}) \\ \end{aligned}.$$.

**Table 2:** Information on the 8 immune-related gene pairs (IRGPs).

IRG 1	Immune processes	IRG 2	Immune processes	Coefficient
BIRC5	Antimicrobials	BPHL	Antimicrobials	1.139
CCL2	Antimicrobials	OAS1	Antimicrobials	− 0.658
CD19	BCR Signaling Pathway	PI3	BCR Signaling Pathway	− 0.461
CD3G	TCR signaling Pathway	IL7	Cytokines	− 0.557
DKK1	Cytokines	IKBKB	TCR signaling Pathway	0.723
F2RL1	Antimicrobials	LTB	Cytokines	0.448
PIK3CD	BCR Signaling Pathway	S100A2	Antimicrobials	− 0.428
SERPIND1	Antimicrobials	VEGFC	Cytokines	− 0.606

*IRGPs* immune-related gene pairs, *IRG* immune-related gene.

### Validation and evaluation of the prognostic IRGPs sigture

**Figure 2:**
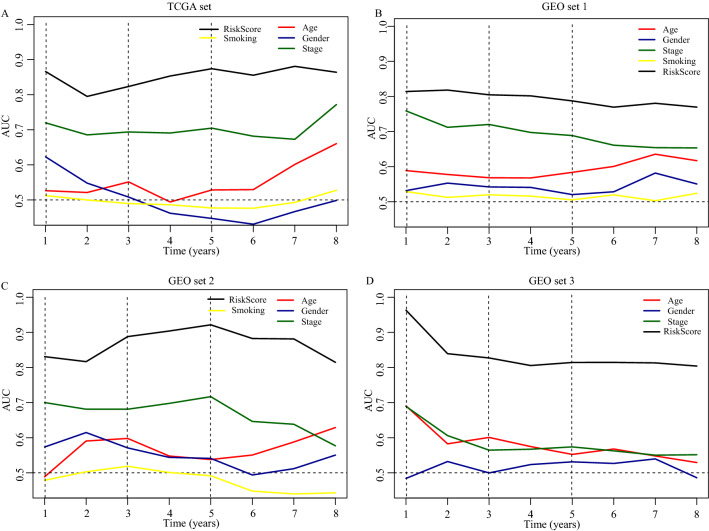
The survival prediction performance of the IRGPs signature. (**A**) The time-dependent receiver operating characteristic curve (ROC) in the TCGA set, (**B**) in the GEO set 1. (**C**) in the GEO set 2. (**D**) in the GEO set 3. TCGA: The Cancer Genome Altas; GEO: Gene Expression Omnibus; AUC: the area under curve of ROC.

In the TCGA set, the “surv_cutpoint” function of the R package ‘Survminer’ was applied for determining the optimal cut-off value of immune risk score, which can best dichotomize patients according to prognosis difference. According to the optimal cut-off risk score: 1.84 (Fig. S1), patients were divided into low- and high-risk groups. Then, we depicted the time-dependent AUC (Fig. 2) and c-index (Fig. S3A) to assess the predictive accuracy and effectiveness of the prognostic IRGPs signature. The AUC for predicting 1- and 3- year OS in the TCGA set was 0.867 and 0.870 (2A; Fig. S2), respectively, with the c-index = 0.873 and 0.804 (Fig. S3A). In the GEO set 1, 2, and 3, the AUC of 1-year OS was 0.819, 0.834, and 0.955, respectively, and of 3-year OS was 0.803, 0.870, and 0.827, respectively (Fig. 2B–D; Fig. S2). All of the AUCs in four sets were significantly higher than the AUCs of clinical indexes (Fig. 2). Besides, stratification analyses demonstrated the stable predictive power of the IRGPs signature in each subgroup (Patients > 50; Fig. S4).

### The IRGPs signature is an independent prognostic factor of overall survival

**Figure 3:**
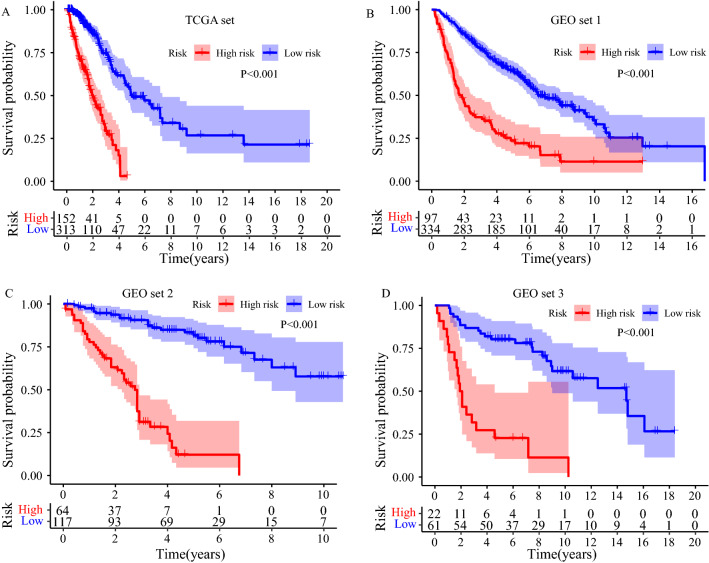
Survival difference between high- and low-risk group (**A**) in the TCGA set, (**B**) in the GEO set 1. (**C**) in the GEO set 2. (**D**) in the GEO set 3.

**Figure 4:**
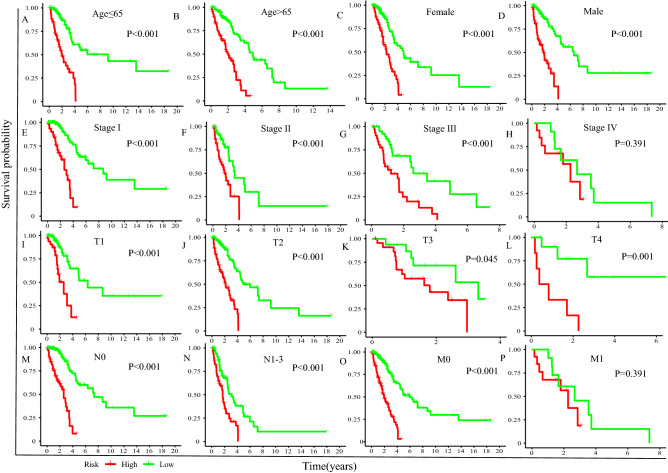
Stratification analyses of overall survival between high- and low-risk patients in different subgroup. (**A**) Age ≤ 65. (**B**) Age > 65. (**C**) Female. (**D**) Male. (**E**) TNM grade I. (**F**) TNM grade II. (**G**) TNM grade III. (**H**) TNM grade IV. (**I**) T1. (**J**) T2. (**K**) T3. (**L**) T4. (**M**) Without lymph node metastasis. (**N**) With lymph node metastasis. (**O**) Without distance metastasis. (**P**) With distance metastasis.

Survival analysis was carried out to compare the survival difference between low- and high-risk groups. All of Kaplan–Meier plots in four sets demonstrated that high-risk LUAD patients exhibited poorer prognosis than low-risk LUAD patients (TCGA set: *P* < 0.001, Fig. 3A; GEO set 1: *P* < 0.001, Fig. 3B; GEO set 2: *P* < 0.001, Fig. 3C; GEO set 3: *P* < 0.001, Fig. 3D). Furthermore, stratification analyses showed the clinical outcome of high-risk LUAD patients in each stratum of age, gender, TNM stage, tumor size, lymph node metastasis, and distance metastasis was poorer than that of low-risk patients except in subgroup patients within Stage IV and with distance metastasis (Fig. 4).

**Figure 5:**
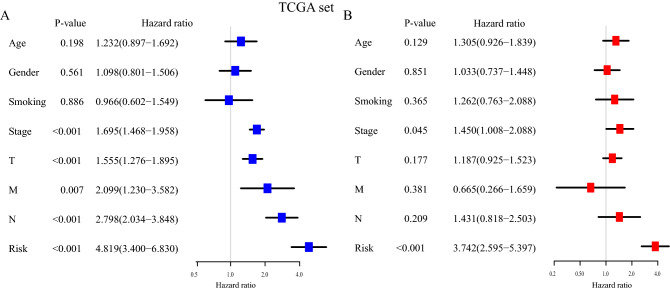
The IRGPs signature is an independent prognostic factor for the prognosis of LUAD patients. (**A**) The result of univariable Cox regression analysis in the TCGA set. (**B**) The result of multivariable Cox regression analysis in the TCGA set.

Then, we took advantage of the univariate and multivariate Cox regression model to compare the immune risk score with clinical parameters (age, gender, smoking, histologic grade, TNM grade, tumor size, lymph node metastasis, and distance metastasis). The univariable Cox regression analysis in the TCGA set indicated that the risk score was an important factor for patients’ prognosis (TCGA set: HR = 4.819, 95% CI [3.400, 6.830], *P* < 0.001, Fig. 5A), in line with the results in the GEO set 1, 2, and 3 (GEO set 1: HR = 3.178, 95% CI [2.405, 4.200], *P* < 0.001, Fig. S5A; GEO set 2: HR = 9.598, 95% CI [5.403, 17.050], *P* < 0.001, Fig. S5B; GEO set 3: HR = 6.632, 95% CI [3.380, 13.014], *P* < 0.001, Fig. S5C). Moreover, the multivariable Cox regression in the TCGA set demonstrated that the risk score was an independent predictive indicator for the OS of LUAD patients (TCGA set: HR = 3.742, 95% CI [2.595, 5.397], *P* < 0.001, Fig. 5B). It was confirmed in the GEO set 1, 2, and 3 (GEO set 1: HR = 2.473, 95% CI [1.789, 3.436], *P* < 0.001, Fig. S5D; GEO set 2: HR = 3.524, 95% CI [2.496, 4.975], *P* < 0.001, Fig. S5E; GEO set 3: HR = 8.446, 95% CI [4.649, 15.344], *P* < 0.001, Fig. S5F).

### Correlation between the IRGPs signature and clinical characteristics

**Figure 6:**
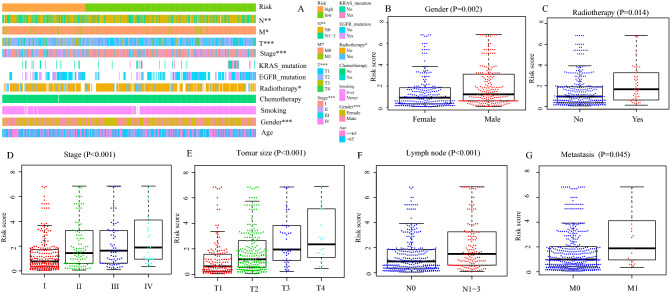
Correlation between the IRGPs signature and clinical characteristics. (**A**) Heat map for the distribution of clinicopathological features between high- and low- risk group. (**B**) The difference of risk score between different gender, (**C**) between patients with and without radiotherapy, (**D**) among different TNM grades, (**E**) among different tumor size, (**F**) between with and without lymph node metastasis, (**G**) between with and without distance metastasis. *P < 0.05, **P < 0.01, ***P < 0.001.

Next, we evaluated the correlation between the IRGPs signature and clinical characteristics (age, gender, smoking, EGFR mutation, KARS mutation, radiotherapy, chemotherapy, TNM stage, tumor size, lymph node metastasis, and distant metastasis). As shown in Fig. 6A, between low- and high-risk group, the distribution of gender (*P* = 0.001), TNM stage (*P* < 0.001), tumor size (*P* < 0.001), lymph node metastasis (*P* < 0.001), distant metastasis (*P* = 0.037), and patients with or without radiotherapy (*P* = 0.028) was significantly different. Meanwhile, compared with female patients, the immune risk score in male patients was significantly increased (*P* = 0.002, Fig. 6B). A similar phenomenon was observed in patients with radiotherapy (*P* = 0.014, Fig. 6C), lymph node metastasis (*P* < 0.001, Fig. 6F), and distance metastasis (*P* = 0.045, Fig. 6G). In addition, with the increase of TNM grade (*P* < 0.001, Fig. 6D) and tumor size (*P* < 0.001, Fig. 6E), the immune risk score was also increased. There was no difference in risk score between patients aged ≤ 65 or > 65, with or without EGFR mutation, with or without EGFR mutation, smoking or non-smoking, and with or without chemotherapy.

### Relationship between the IRGPs and tumor-infiltrating immune cells (TIICs)

**Figure 7:**
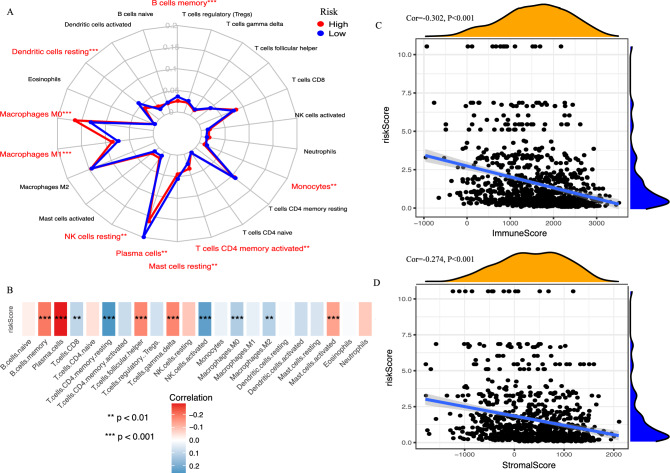
Relationship between the IRGPs signature and tumor-infiltrating immune cells (TIICs). (**A**) The difference of 22 TIICs between high- and low- risk group. (**B**) The Spearman correlation analysis revealed the relationship of immune risk scores to 22 TIICs. The Spearman correlation analysis revealed the relationship of immune risk scores to (**C**) immune scores and (**D**) stromal scores. *P < 0.05, **P < 0.01, ***P < 0.001.

The most abundant TIICs were Macrophages (M0, M1, M2) (32.98%), followed by Plasma cells (17.23%) and resting memory CD4 T cells (9.70%). The proportions of Macrophages M0 (*P* < 0.001), Macrophages M1 (*P* < 0.001), activated memory CD4 T cells (*P* = 0.002), and resting NK cells (*P* = 0.003) were significantly increased in the high-risk group, whereas, the proportions of memory B cells (*P* < 0.001), Plasma cells (*P* = 0.008), Monocytes (*P* = 0.006), resting Dendritic cells (*P* < 0.001), and resting Mast cells (*P* = 0.005) were significantly decreased (Fig. 7A). Furthermore, Spearman correlation analysis showed the immune risk score was negatively correlated with the proportion of Plasma cells (cor =  −0.286, *P* < 0.001) and memory B cells (cor =  −0.201, *P* < 0.001), and positively correlated with the percentage of resting memory CD4 T cells (cor = 0.257, *P* < 0.001) and activated NK cells (cor = 0.235, *P* < 0.001) (Fig. 7B). Then, we calculated immune and stromal scores with the ESTIMATE algorithm, and found that the immune risk score was also highly related to immune scores (cor =  − 0.302, *P* < 0.001, Fig. 7C) and stromal scores (cor =  −0.274, *P* < 0.001, Fig. 7D).

### Expression profile of immunomodulators

In the present study, we quantified 11 immunomodulators (CTLA4, ICOS, ICOSLG, IFN- γ, LAG3, NKG2A, PD − 1, PD − L1, TIGIT, TIM3, and VISTA). The expressions of CTLA4 (*P* < 0.001), ICOS (*P* < 0.001), PD − 1 (*P* = 0.002), TIGIT (*P* < 0.001), TIM3 (*P* = 0.001), and VISTA (*P* = 0.001) were significantly up-regulated in the low-risk group compared with that in the high- risk group (Fig. S6).

### Gene set enrichment analysis (GSEA)

**Figure 8:**
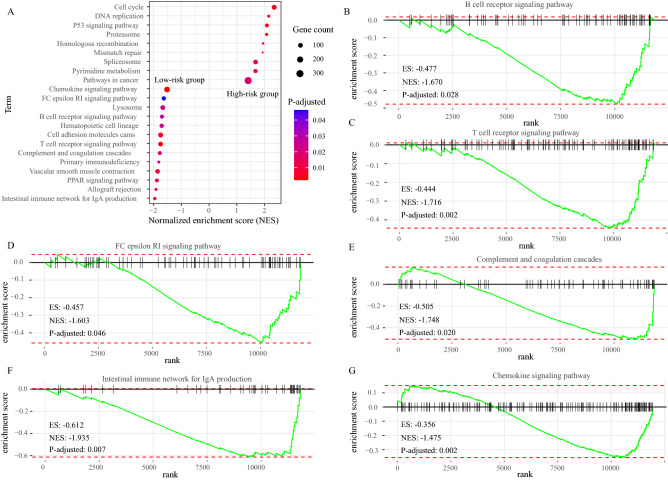
Gene set enrichment analysis (GSEA) between high and low immune risk groups. (**A**) 22 KEGG pathway-related gene sets, including 9 pathways in high0resk group and 13 pathways in the low-risk group. The X axis represented NES. The nodes represented pathways, the colour represented *P*-adjusted, the size represented gene counts. (**B–G**) 6 immune-related pathways enriched in low-risk group. *NES* normalized enrichment score; *ES* enrichment score.

To explore the basic biological mechanisms of the IRGPs signature, we carried out GSEA analysis. A total of 22 KEGG pathways were identified between the high-risk and low-risk groups, including 9 pathways in the high-risk group and 13 pathways in the low-risk group (Fig. 8A; Table S1). Of note, in the low-risk group, various immune-related KEGG pathways were enriched, such as “B cell receptor signaling pathway” (Normalized enrichment score (NES): − 1.670, *P*-adjusted: 0.028; Fig. 8B), “T cell receptor signaling pathway” (NES: − 1.716, *P*-adjusted: 0.002; Fig. 8C), “FC epsilon RI signaling pathway” (NES: − 1.603, *P*-adjusted: 0.046; Fig. 8D), “Complement and coagulation cascades” (NES: − 1.748, *P*-adjusted: 0.020; Fig. 8E), “Intestinal immune network for IgA production” (NES: − 1.935, *P*-adjusted: 0.007; Fig. 8F), and “Chemokine signaling pathway” (NES: − 1.475, *P*-adjusted: 0.002; Fig. 8G). In addition, 9 pathways were significantly enriched in the high-risk group, which were highly associated with the tumorigenesis and development of cancers (Fig. 8A, Table S1).

### Construction of nomogram for predicting 1- and 3-year survival probability

Previously, the multivariate Cox regression analysis identified TNM stage and risk score were independent OS-related predictors (Fig. 5). Therefore, we used TNM stage and risk score to develop a prognostic nomogram for predicting 1- and 3-year survival probability in LUAD patients. The nomogram was presented in Fig. 1D. The AUCs of the nomogram reached 0.905 at 1-year, and 0.901 at 3-year (Fig. S3B).

## Discussion

In the current study, we collected the immune-related gene matrix from the TCGA database to construct immune-related gene pairs (IRGPs). A total of 12,334 IRGPs were paired, and a prognostic IRGPs signature based on 8 IRGPs was established with multivariate Cox regression analysis. According to the cut-off immune risk score, LUAD patients were divided into high- and low-risk groups. Survival analysis demonstrated that high-risk patients predicted poorer clinical outcomes. Moreover, the result of multivariate Cox regression analysis showed that the immune risk score was an independent prognostic factor for LUAD patients. Then, we evaluated the predictive effectiveness and accuracy of the prognostic IRGPs signature for 1- and 3-year OS and validated this finding. The AUC of the signature in the TCGA set for predicting 1- and 3-year OS was 0.867 and 0.870, respectively, which was significantly higher than the AUC of clinical parameters, such as TNM stage. Similar results was observed in the GEO set 1, 2, and 3. In addition, the c-index in the TCGA set for 1- and 3-year OS was 0.873 and 0.804, respectively, in line with the c-index in the GEO set 1, 2, and 3 (Fig. S3A). All data suggested that the prognostic IRGPs signature was stable and reliable, and suitable for estimating 1- and 3-year survival probability of LUAD patients.

In recent years, several prognostic signatures based on RNA-seq or microarray expression have been established for exploring prognosis-related biomarkers and predicting the 1- and/or 3-year OS of LUAD. For example, a study built an immune signature for 1- and 3-year survival rate of LUAD^14^. The AUC for 1- and 3-year of the immune signature in the training cohort was 0.70 and 0.68, respectively, all of which were inferior to the AUCs in this study. Similarly, a study reported an immune-related signature^15^, which AUC of 1-year (0.78) and 3-year (0.76) OS was also lower than that of this study. In addition, a study reported a glycolysis-related gene prognostic signature with the AUC = 0.72^16^. And, a previous study developed an autophagy-related gene prognostic signature with the AUC = 0.615^17^. Both the AUC of these two studies were inferior to that of the IRGPs signature. Moreover, those predicting signatures were constructed directly with the expression level of genes based on microarray expression and RNA-seq. And, due to the technical bias and biological heterogeneity, it is difficult to standardize gene expression profiles produced by various platforms when using other datasets to validate predicting signatures. Additionally, problems such as over-fitting on small sample training data-sets and lack of enough verification datasets often occurred. In this study, we collected 465 LUAD cases from the TCGA database to develop an IRGPs signature. Meanwhile, for avoiding over-fitting, we used three independent datasets including 695 cases to validate the signature. The AUC and c-index in the TCGA set and the other three independent sets were similar. Additionally, the method of gene pair was based on a relative ranking of gene expression level to make pairwise comparison and generate the score in the same patient, which could eliminate the shortcomings, such as the batch effect of different platforms^12,13,18^.

Nowadays, emerging studies show the tumor microenvironment (TME) is critical for the initiation, progression, and metastasis of cancers, and therapy targeting the TME seems to be an encouraging method to overthrow therapeutic escape issues^19,20^. In this study, we also calculated the proportions of 22 TIICs in TME of LUAD and found that macrophages were the most abundant immune cell, which was in line with the previous findings. In addition, in high-risk patients, the proportions of macrophages M1 were significantly increased. However, Spearman correlation analysis revealed that there was no noteworthy relationship between the immune risk score and the proportion of Macrophages M1. However, Spearman correlation analysis demonstrated that the immune risk score was highly related to Plasma cells and memory B cells, which were favorable prognostic factors for LUAD. Previous researches demonstrated accumulating memory B cells in TME was strongly correlated with favorable clinical outcomes in various tumors^20^. In TME, B cells could produce antibodies and present antigens to regulate innate immunity and promote antigen-specific immune responses to repress tumor development^21–23^. Besides, studies reported that high tumor-infiltrating plasma cells was a prognostic marker in NSCLC, and predicted better clinical outcomes^24,25^, which was in line with the result in this study. Meanwhile, GSEA revealed that immune-related pathways were mainly enriched in the low-risk group. Those results demonstrated the immunity between high- and low-risk patients was dissimilar, and in low-risk patients, the immune response was more active, which may contribute to increased survival time in LUAD patients.

Although the prognostic IRGPs signature showed a well predictive accuracy and effectiveness for LUAD patients in this study, there are still some limitations that needed to be addressed. Firstly, our research was a retrospective study, and all cases were retrospective samples. Hence, validation of prospective samples was still needed. And, the enrolled patients mainly consisted of white, and the predictive accuracy and effectiveness in other races remained explored. Secondly, owing to all samples were collected from the public database, the potential selection bias could not be excluded, and some clinical information such as KRAS mutation, EGFR mutation, immunotherapy and so on were missing, which may lead to information bias. Thirdly, herein, we performed stratification analyses and determined the significant survival difference between the low- and high-risk groups and the stable and reliable predictive power of the IRGPs signature in each subgroup. However, due to lacking information of therapies like surgery, targeted therapy, and immunotherapy in most patients, we could not homogenize the treatment and evaluate the predictive effectiveness and accuracy of the IRGPs signature in patients with surgery, targeted therapy, and immunotherapy. It may bring biased prognosis predictions. Fourthly, the signature was constructed based on microarray expression and RNA-seq data, which is costly and time-consuming. And, it lacked validation using PCR or immunohistochemistry. Finally, evidences illustrated the IRGPs signature was highly related to a number of TIICs, immunomodulators, and immune-related pathways, hinting the IRGPs signature may predict the clinical benefit of immunotherapy and screen out patients who benefit from immunotherapy. However, there was no experimental data from our laboratory to testify the finding and explore the mechanism in depth in this study. Hence, further investigation is demanded to examine the discovery of this research both in vitro and in vivo.

Take together, in the current study, we developed a prognostic IRGPs signature with 8 immune-related gene pairs for predicting 1- and 3- year overall survival in LUAD. This signature will be an available predictive tool to identify patients who might benefit from immunotherapy and provide a convenient tool for risk assessment and prognosis assessment.

## Methods

### Patient data sets

The FPKM level gene expression matrixes were taken from the TCGA database. The raw data of mRNA expression matrix of GSE68465, GSE41271, and GSE30219 were downloaded from the GEO database, and normalized with the MAS5.0 method using the “affy” and “lumi” package in R 3.6.3 (https://www.r-project.org). The platform for GSE68465 was GPL96 (Affymetrix Human Genome U133A Array), for GSE41271 was GPL6884 (Illumina HumanWG-6 v3.0 expression beadchip), and for GSE30219 was GPL570 (Affymetrix Human Genome U133 Plus 2.0 Array). In addition, relevant clinical characteristics of patients were also collected, and patients lacking survival time and survival state would be removed, thanks to they were not representative for analyzing prognostic factors.

### Construction of a prognostic IRGPs signature

The IRGPs signature was constructed as described by a previous study^12^. The method for gene pair was based on a relative ranking of gene expression level. One immune-related gene (IRG) paired with another IRG randomly to construct a gene pair. In a specific sample, the expression levels of two genes in a gene pair were performed paired comparison to generate a score for the gene pair. In a specific IRGP, if the first IRG expression level was lower than the second IRG expression level, the score of this IRGP was 0; otherwise, the score was 1. We constructed IRGPs in four sets and screened out overlapping IRGRs. Then, in the TCGA set, we performed univariate Cox regression analysis and LASSO regression analysis to screen out OS-related IRGPs. Finally, multivariate Cox regression analysis was carried out to identify top OS-related IRGPs and to establish a prognostic IRGPs signature and an immune risk score formula.

$$ {\text{Risk}}\,{\text{score}} = \mathop \sum \limits_{i = 1}^{n} coeffcient*Score\,of\,IRGP\left( i \right) $$

### Validation and evaluation of the prognostic IRGPs signature

With the above risk score formula, the immune risk score of patients was calculated. The optimal cut-off value of the immune risk score was determined with the R package ‘Survminer’ and classified patients into low- and high-risk score groups. The AUC and c-index were calculated to assess the predictive accuracy and effectiveness of this prognostic IRGPs signature.

### Correlation between the IRGPs signature and TIICs

The CIBERSORT algorithm is a novel accurate way that can determine 22 TIICs simultaneously in TME^26^. With this algorithm, we quantified the proportions of 22 TIICs in all samples. CIBERSORT *P* < 0.05 was considered as cut-off value. ESTIMATE algorithm is a novel tool based on a large scale of gene expression profile, and could be used for estimating the level of infiltrating immune and stromal cells by calculating immune and stromal scores^27^. Herein, we applied the ESTIMATE algorithm to infer immune and stromal scores of each sample with the BiocManager package: estimate in R^27^. Meanwhile, several key immunomodulators were also quantified.

### GSEA

To determine the biological processes and signaling pathways altered by the IRGPs signature, GSEA was performed with the BiocManager package: fgsea. *P*-adjusted < 0.05 was set as the cut-off value.

### Statistical analysis

All statistical analyses were performed with R 3.6.3 software. For categorical data, the chi-square test was performed to compare the differences among different groups, whereas, for measurement data, the t-test or one-way ANOVA was used. Survival curves were performed by the Kaplan–Meier method, and survival rates were compared with the log-rank test. Moreover, the univariate Cox regression analysis and multivariate Cox regression analysis were also performed to identify independent prognostic factors. The relationships of 22 TIICs as well as immune and stromal scores to the immune risk scores were investigated with the Spearman correlation analysis.

### Ethical approval and consent to participate

All data in this study were collected from public databases: TCGA and GEO. This article does not contain any studies with patients or animals performed by any of the authors.

## Supplementary Information

Supplementary Information.

## Data Availability

The data that support the findings of this study are openly available in the TCGA database: http://cancergenome.nih.gov/ and GEO database: https://www.ncbi.nlm.nih.gov/geo/.
