# Supplementary material for: Development of an immune-related gene pairs signature for predicting clinical outcome in lung adenocarcinoma
Source: Sci Rep. 2021 Feb 11;11:3611. doi: 10.1038/s41598-021-83120-4 (PMC7878883; doi:10.1038/s41598-021-83120-4)
Supplement: Supplementary file 1 — Supplementary Information. [file 41598_2021_83120_MOESM1_ESM.pdf]

Development of an immune-related gene pairs signature for predicting clinical outcome in lung adenocarcinoma

Chunlei Wu[1], Quanteng Hu[1], & Dehua Ma[*]

Department of Thoracic Surgery, Taizhou Hospital, Taizhou, Zhejiang, China

1 These authors contributed equally to this work.

*Correspondence: Dehua Ma, Department of Thoracic Surgery, Taizhou Hospital, No. 150 Ximen street, Linhai, Taizhou, Zhejiang, China.

Email:madh_tzyy@sina.com

Table S1. 22 pathways were enriched between high and low immune risk groups with GSEA.

Group	Pathway	ES	NES	Gene count	P-adjusted
High-risk group	Cell cycle	0.636	2.380	114	0.005
	P53 signaling pathway	0.624	2.111	60	0.005
	Proteasome	0.677	2.094	39	0.005
	Homologous recombination	0.697	1.961	25	0.005
	DNA replication	0.720	2.168	34	0.005
	Mismatch repair	0.714	1.944	22	0.007
	Spliceosome	0.467	1.709	100	0.012
	Pyrimidine metabolism	0.485	1.702	76	0.016
	Pathways in cancer	0.345	1.460	303	0.018
Low-risk group	T cell receptor signaling pathway	-0.444	-1.716	103	0.002
	Chemokine signaling pathway	-0.356	-1.475	165	0.002
	Intestinal immune network for IgA production	-0.612	-1.935	41	0.007
	Cell adhesion molecules cams	-0.453	-1.711	115	0.007
	Allograft rejection	-0.628	-1.894	33	0.009
	Vascular smooth muscle contraction	-0.497	-1.824	95	0.009
	PPAR signaling pathway	-0.542	-1.854	61	0.012
	Complement and coagulation cascades	-0.505	-1.748	66	0.020
	Hematopoietic cell lineage	-0.467	-1.677	82	0.024
	Lysosome	-0.439	-1.636	105	0.024
	Primary immunodeficiency	-0.595	-1.793	33	0.024
	B cell receptor signaling pathway	-0.477	-1.670	71	0.028
	FC epsilon RI signaling pathway	-0.457	-1.603	72	0.046

Abbreviations: GSEA: Gene set enrichment analysis; ES: enrichment score; NES: Normalized enrichment score.

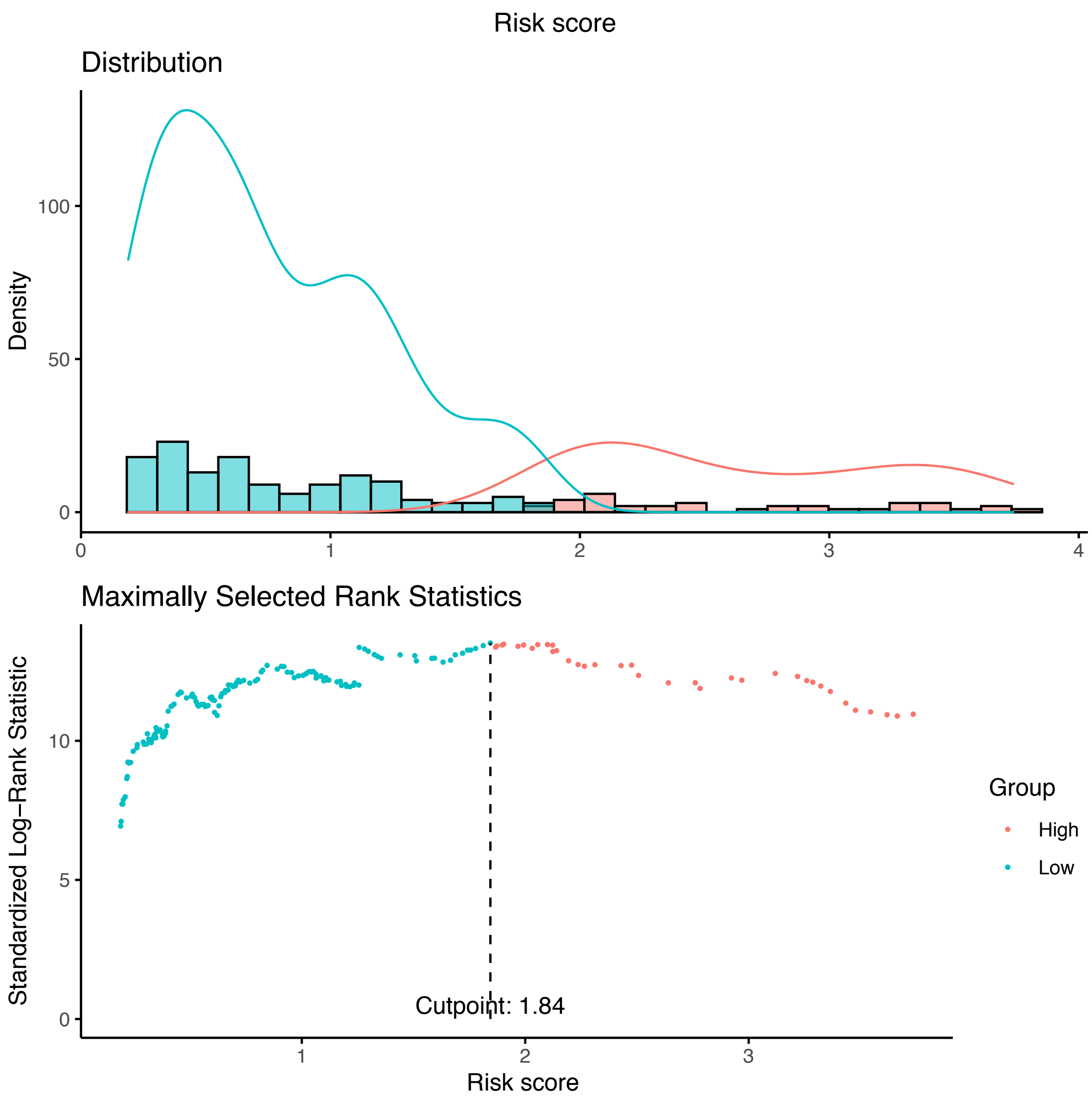

Figure S1. Determination of optimal cut-off value of immune risk score in TCGA set with R package 'Survminer'.

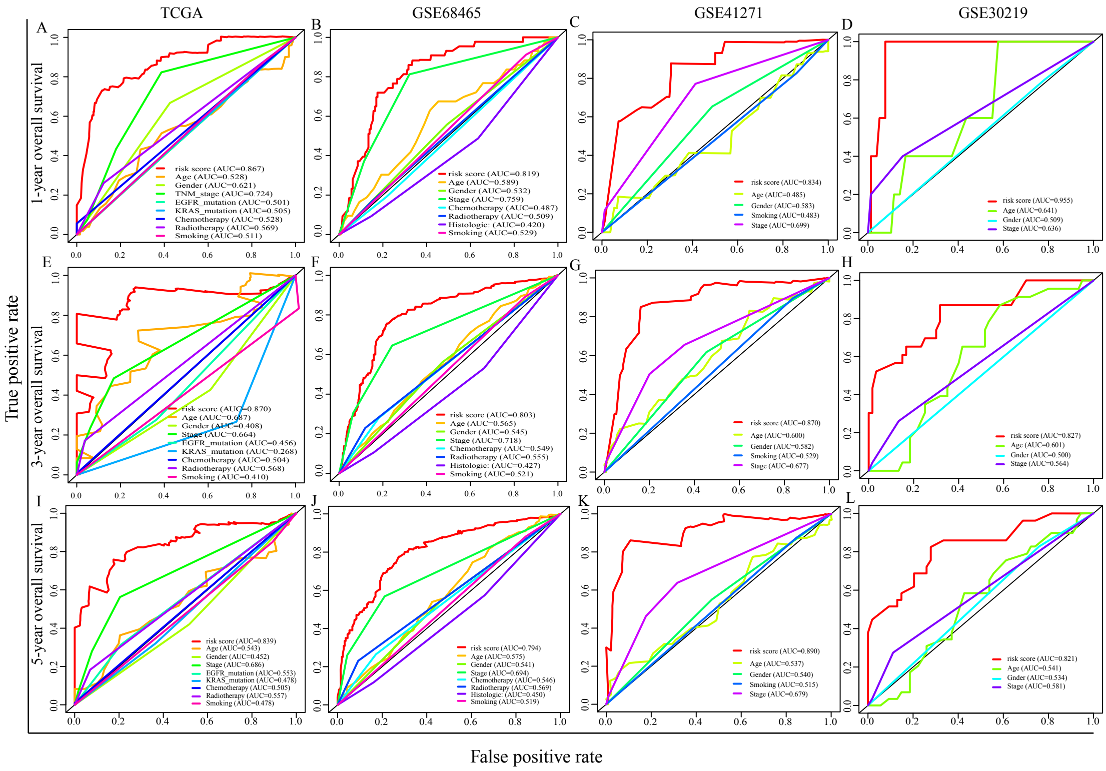

Figure S2. Evaluation of IRGPs signature. (A) The area under the receiver operating characteristic (ROC) curve (AUC) for 1-year overall survival of LUAD patients in TCGA set, (B) in GEO set 1, (C) in GEO set 2, (D) in GEO set 3. (E) The AUC for 3-year overall survival in TCGA set, (F) in GEO set 1, (G) in GEO set 2, (H) in GEO set 3. (I) The AUC for 5-year overall survival in TCGA set, (J) in GEO set 1, (K) in GEO set 2, (L) in GEO set 3.

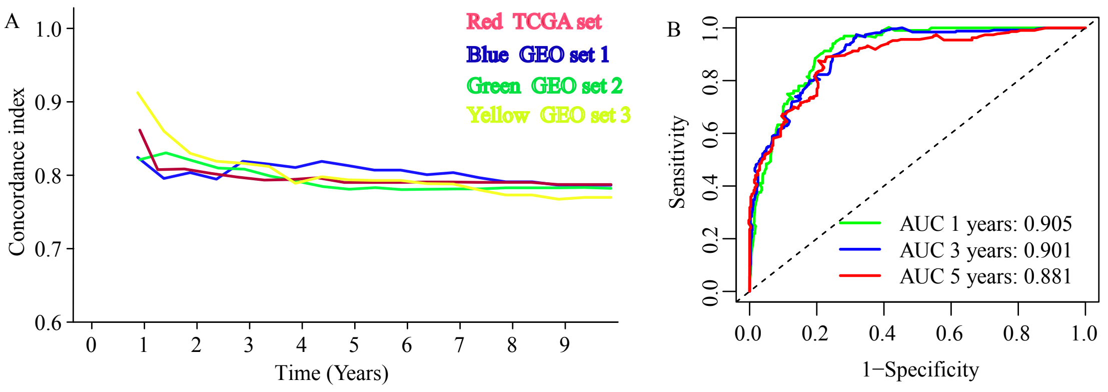

Figure S3. (A) Time dependent c-index in four sets. (B) The AUCs of the nomogram for predicting 1- and 3-year survival probability.

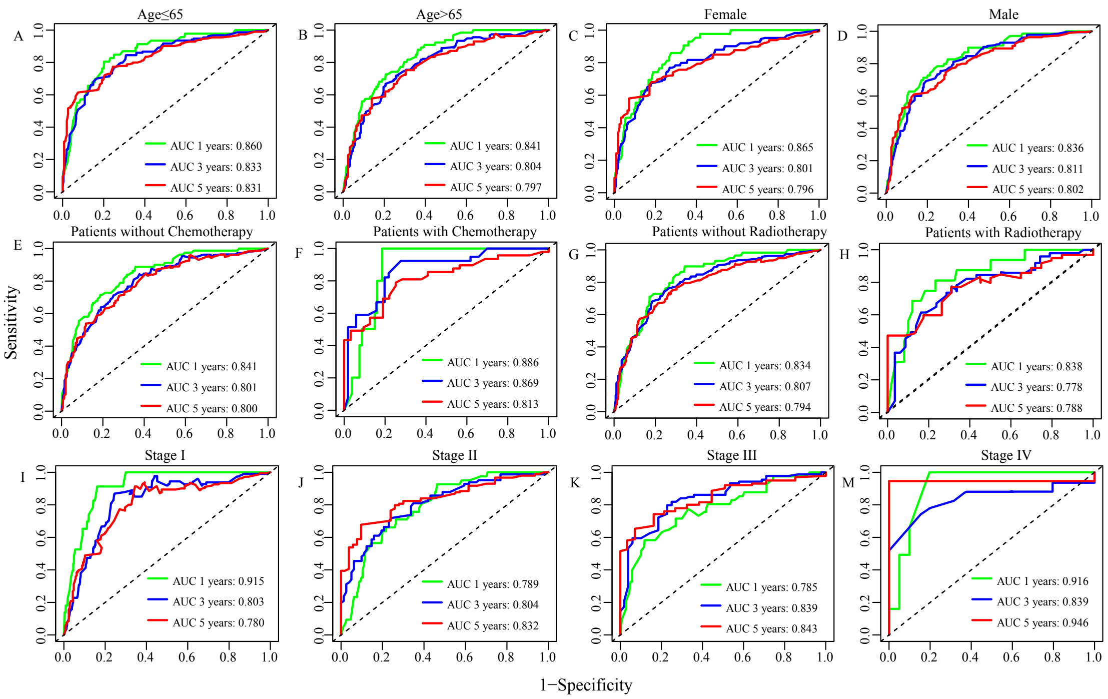

Figure S4. Stratification analyses of the AUCs of the IRGPs signature in each subgroup. (A) Age≤65. (B) Age>65. (C) Female. (D) Male. (E) Patients without Chemotherapy. (F) Patients with Chemotherapy. (G) Patients without Radiotherapy. (H) Patients without Radiotherapy. (I) TNM stage I. (J) TNM stage II. (K) TNM stage III. (L) TNM stage IV.

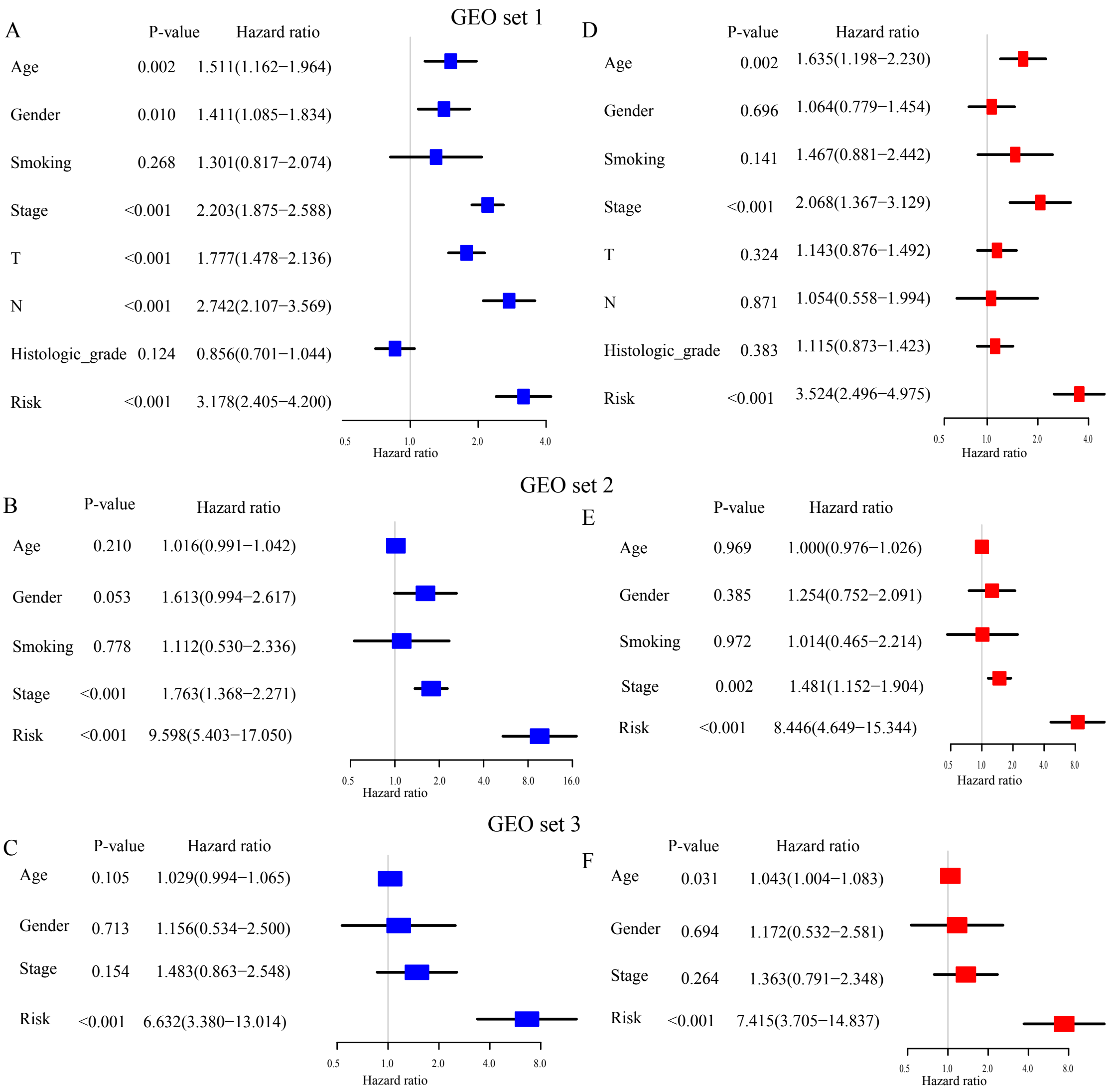

Figure S5. Validation of the IRGPs signature as an independent prognostic factor for the prognosis of LUAD patients. (A) The result of univariable Cox regression analysis in the GEO set 1. (B) in the GEO set 2. (C) in the GEO set 3. (D) The result of multivariable Cox regression analysis in the GEO set 1. (E) in the GEO set 2. (F) in the GEO set 3.

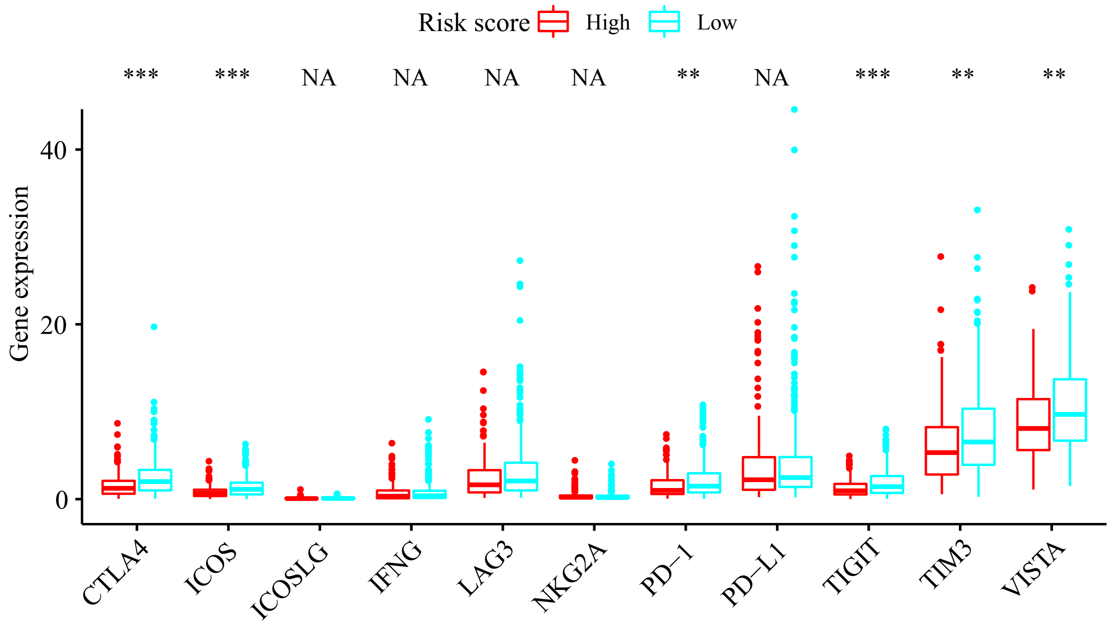

Figure S6. The difference of distribution of 11 immunomodulators between high- and low- risk LUAD patients. ** P<0.01, ***P<0.001.
